# *Plasmodium berghei* oocysts possess fatty acid synthesis and scavenging routes

**DOI:** 10.1038/s41598-023-39708-z

**Published:** 2023-08-05

**Authors:** Sadia Saeed, Annie Z. Tremp, Johannes T. Dessens

**Affiliations:** Department of Infection Biology, Faculty of Infectious and Tropical Diseases, London School of Hygiene & Tropical Medicine, Keppel Street, London, WC1E 7HT UK

**Keywords:** Cell biology, Microbiology

## Abstract

Malaria parasites carry out fatty acid synthesis (FAS) in their apicoplast organelle via a bacterially related (type II) enzymatic pathway. In the vertebrate host, exoerythrocytic *Plasmodium* stages rely on FAS, whereas intraerythrocytic stages depend on scavenging FA from their environment. In the mosquito, *P. falciparum* oocysts express and rely on FAS enzymes for sporozoite formation, but *P. yoelii* oocysts do not express, nor depend on, FAS enzymes and thus rely on FA scavenging to support sporogony. In *P. berghei*, FAS enzymes are similarly expendable for sporogony, indicating it conforms to the *P. yoelii* scenario. We show here that *P. berghei*, unexpectedly, expresses FAS enzymes throughout oocyst development. These findings indicate that *P. berghei* can employ FAS alongside FA scavenging to maximise sporogony and transmission, and is more similar to *P. falciparum* than previously assumed with respect to FA acquisition by the oocyst. The ability of oocysts to switch between FAS and scavenging could be an important factor in the non-competitive relationship of resource exploitation between *Plasmodium* parasites and their mosquito vectors, which shapes parasite virulence both in the insect and vertebrate.

## Introduction

Malaria remains a life-threatening infectious disease that is caused by infection with apicomplexan parasites of the genus *Plasmodium*, with *P. falciparum* the deadliest among several human malaria parasite species. Clinical symptoms are caused by blood stage (intraerythrocytic) asexual parasites, a small percentage of which develop into sexual stage precursor cells (gametocytes) that facilitate transmission by mosquitoes. Malaria parasite transmission begins with the uptake of male and female gametocytes with the blood meal by a female mosquito. The main developmental steps that take place inside the insect are: (i) gametogenesis and fertilisation in the midgut lumen; (ii) transformation of the zygotes into elongated motile forms termed ookinetes; (iii) crossing of the midgut epithelium by the ookinetes, followed by their transformation into young oocysts; (iv) growth and division of the oocysts to generate thousands of sporozoites, a process called sporogony; (v) sporozoite egress from the oocyst and their colonisation of the mosquito salivary glands. Following a successful sporozoite-infected mosquito bite, liver stage (exoerythrocytic) asexual parasites develop from which new blood stage infections are initiated to complete the *Plasmodium* life cycle.

**Figure 1:**
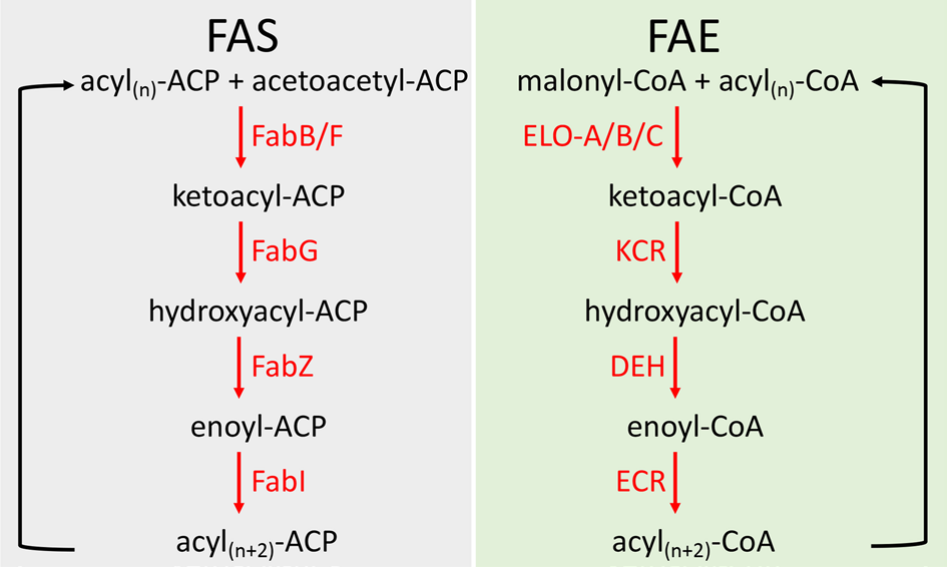
Enzymatic pathways involved in fatty acid synthesis (FAS) and elongation (FAE) in *Plasmodium*. Enzymes involved in catalysis are shown in red font. FabB/F, acyl-ACP elongase; FabG, ketoacyl-ACP reductase; FabZ, hydroxyacyl-ACP dehydratase; FabI, enoyl-ACP reductase; ELO-A/B/C, acyl-CoA elongase A/B/C; KCR, ketoacyl-CoA reductase; DEH, hydroxyacyl-CoA dehydratase; ECR, enoyl-CoA reductase.

Fatty acids (FA) are essential cell components required for the biosynthesis of cellular membranes and signalling molecules. Malaria parasites acquire FA both by de novo synthesis and by scavenging/uptake from their environments. Short- to medium-chain fatty acid synthesis (FAS) is carried out in the apicoplast organelle, a relic chloroplast, and additional FA elongation (FAE) to generate long-chain FA is conducted in the endoplasmic reticulum (ER) membranes, using two parallel and functionally related enzymatic pathways (Fig. 1)^1^. Central to FAS is a four step enzymatic cycle that involves: (i) condensation of acyl-acyl carrier protein (acyl-ACP) with acetoacetyl-ACP, catalysed by acyl-ACP elongase (FabB/F, PBANKA_0823800); (ii) reduction of ketoacyl-ACP, catalysed by ketoacyl-ACP reductase (FabG, PBANKA_0823800); (iii) dehydration of hydroxyacyl-ACP, catalysed by hydroxyacyl-ACP dehydratase (FabZ, PBANKA_1338200); (iv) reduction of enoyl-ACP, catalysed by enoyl-ACP reductase (FabI, PBANKA_1229800) (Fig. 1). FAE uses a similar enzymatic pathway conducted by a different set of proteins, and employing coenzyme A (CoA) instead of ACP as the acyl carrier (Fig. 1).

For malaria parasites, membrane synthesis is especially important for the multiplying phases of the life cycle, *i.e.* during schizogony in the vertebrate host, and during sporogony in the mosquito vector. *Plasmodium* intraerythrocytic stages do not express FAS enzymes^2,3^ and knockout of FAS enzyme-encoding genes shows they are redundant for blood stage parasite development^2–4^. Thus, blood stages must rely on FA scavenging from their environment for their development. In sharp contrast, pre-erythrocytic parasite stages express FAS enzymes, and their depletion results in developmental arrest, demonstrating that the liver stages depend on FAS for their development^1–4^. The situation in the mosquito is more complex and studies have revealed differences between parasite species particularly during sporogony in the oocyst. In the human malaria parasite *P. falciparum*, FabI is expressed in oocysts and genetic depletion of FabI or FabB/F adversely affected development of mature oocysts and abolished sporozoite formation^4^. These observations show that *P. falciparum* oocysts rely on FAS for normal differentiation. By contrast, FAS enzyme expression was not detected in oocysts of the rodent malaria parasite species *P. yoelii* and, consistent with this expression pattern, depletion of FabB/F or FabZ did not impact oocyst or sporozoite development^3^. Accordingly, *P. yoelii* oocysts appear to rely on FA scavenging to support normal oocyst development and sporogony.

While *P. yoelii* and *P. falciparum* oocysts clearly use different strategies for FA acquisition based mainly on scavenging and synthesis, respectively, there is circumstantial evidence to suggest that *P. falciparum* oocysts can use FA scavenging alongside FAS: *P. falciparum* oocyst growth and sporozoite formation are sensitive to artificially lowered lipid levels in the insect achieved via RNAi knockdown of the major mosquito lipid transporter lipophorin^5–7^. Furthermore, increasing mosquito haemolymph lipid levels through extra blood feeding after the initial infected blood meal significantly promotes *P. falciparum* oocyst growth and maturation^6,8,9^. In another rodent malaria parasite, *P. berghei*, it was shown that FabG null mutant parasites form normal numbers of oocyst and produce sporozoites^1^, pointing to a redundant function of FabG—and hence of of FAS—in the oocyst stage and thus a FA acquisition scenario similar to the closely related species *P. yoelii*. In this paper, however, we show that *P. berghei* oocysts, like those of *P. falciparum*, express apicoplast-resident proteins of the FAS machinery. These findings add support to the notion that FA acquisition by oocysts, at least in some *Plasmodium* species including *P. falciparum*, involves a subtle interplay between synthesis and scavenging to maximise sporogony and transmission.

## Results and discussion

**Figure 2:**
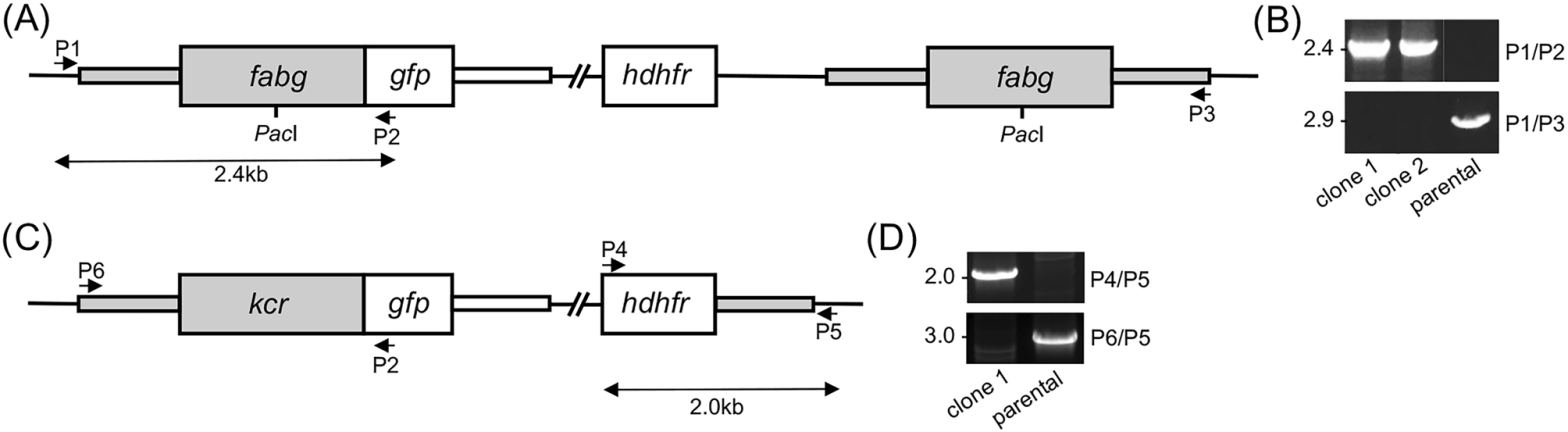
Generation and genotyping of transgenic *P. berghei* parasite lines. (**A**) Schematic diagram of the modified *fabg* alleles in parasite line FabG/GFP generated by single crossover homologous recombination. The *fabg* gene is shown in grey with coding sequence (wide bars) and 5′ and 3′ untranslated regions (narrow bars). Also shown are the *Pac*I restriction site used for single crossover homologous recombination, the *gfp* module, the selectable marker (*hdhfr*), and positions of primers P1-P3 used for diagnostic PCR amplification. Note that this strategy leaves a second, unmodified *fabg* allele. (**B**) PCR with primers P1 and P3 diagnostic for integration of the modified *fabg::gfp* alleles into the *fabg* locus, or with primers P1 and P3 diagnostic for absence of the unmodified parental *fabg* allele. (**C**) Schematic diagram of the modified *kcr* allele in parasite line KCR/GFP generated by double crossover homologous recombination. The *kcr* gene is shown in grey with coding sequence (wide bars) and 5′ and 3′ untranslated regions (narrow bars). Also shown are the *gfp* module, the selectable marker (*hdhfr*), and positions of primers P4-P6 used for diagnostic PCR amplification. (**D**) PCR with primers P4 and P5 diagnostic for integration of the modified *kcr::gfp* alleles into the *kcr* locus, or with primers P6 and P5 diagnostic for absence of the unmodified parental *kcr* allele. See Methods section for primer sequences. Full-size images of the DNA agarose gels are available in the Supplementary Information.

We recently showed that the crystalloid organelle of *P. berghei*, which is found exclusively in the ookinete and young oocyst stages, accommodates the NADPH-generating enzyme NAD(P) transhydrogenase (NTH), which is also present in the downstream sporozoite apicoplast^10^. As the FAS enzyme FabG is NADPH-dependent (Fig. 1) we investigated whether it, like NTH, also located in crystalloids. To do so, a transgenic parasite line expressing FabG fused at its carboxy-terminus to GFP was generated. Diagnostic PCR across the 5′-integration site amplified an approximately 2.4kb fragment of the expected size (Fig. 2), confirming integration of the modified *fabg::gfp* allele into the target locus of the resulting FabG/GFP parasite line. Furthermore, the absence of the parental *fabg* allele in clonal populations of FabG/GFP was confirmed by diagnostic PCR (Fig. 2).

**Figure 3:**
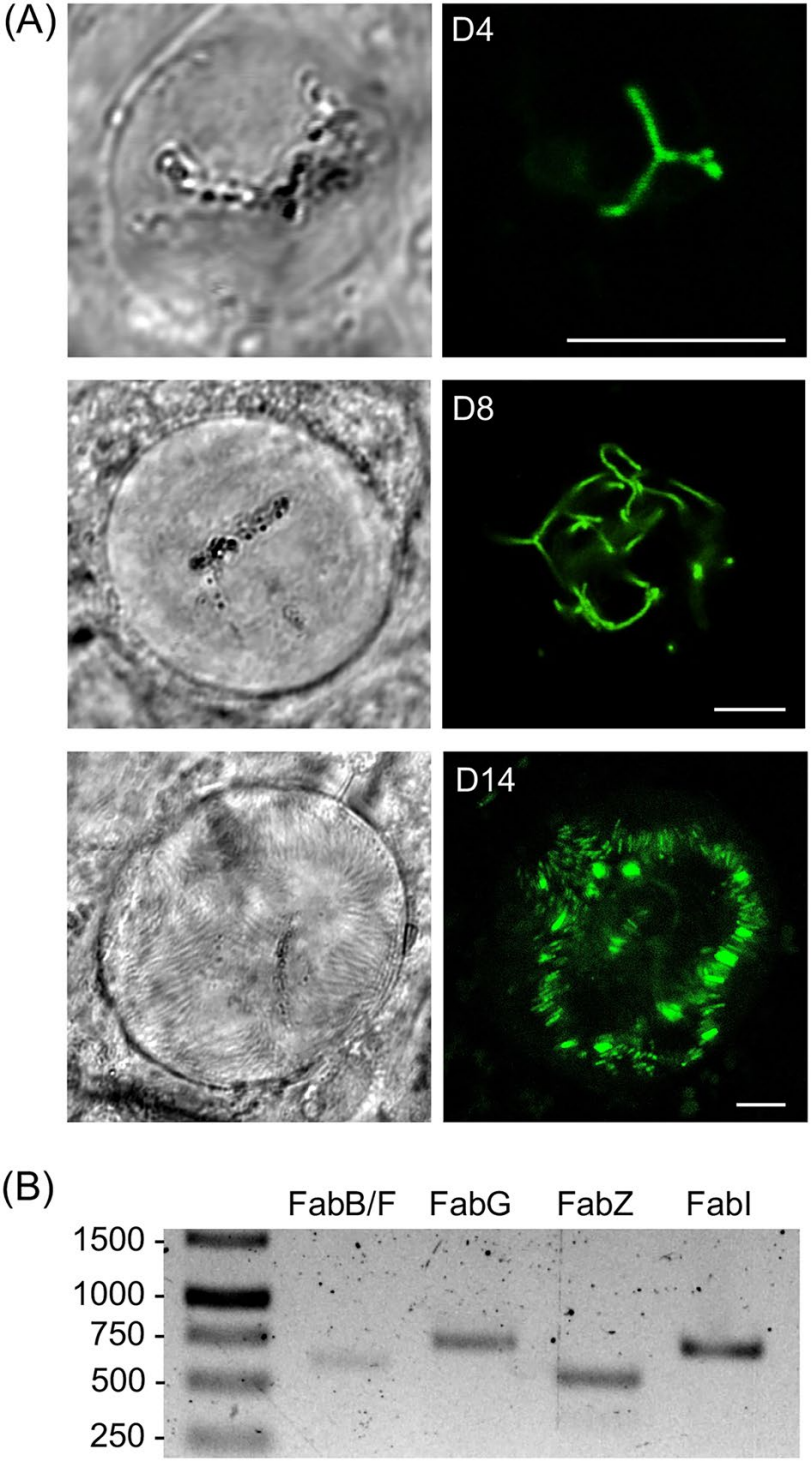
Expression of FAS enzymes in *P. berghei* oocysts. (**A**) Confocal GFP fluorescence and brightfield images of unsporulated (4 and 8 days post-infection) and sporulating (14 days post-infection) oocysts of parasite line FabG/GFP (clone 1). Scale bar 5μm. (**B**) Reverse transcription PCR of unsporulated wildtype oocyst samples with primers specific for mRNA of genes encoding FabB/F, FabG, FabZ and FabI, amplifying 639, 769, 562 and 750bp fragments, respectively. A full-size image of the DNA agarose gel is available in the Supplementary Information.

Assessment of GFP expression by confocal fluorescence microscopy of live FabG/GFP parasites revealed a lack of GFP fluorescence in blood stage parasite, gametes and ookinetes (data not shown) indicating that FabG expression is absent or very low in these life stages. This observation is consistent with transcriptome data reported for the *P. berghei fabg* gene, showing very low transcript levels in blood stage parasites^11^. Surprisingly, however, FabG expression was readily detected in young oocysts (Fig. 3A). Fluorescence was localised in a tubular branched structure reminiscent of the apicoplast (Fig. 3A), in agreement with localisation of FAS in this organelle. FabG::GFP remained apicoplast-localised throughout oocyst development and in sporulated oocysts located in discrete spots, likely reflecting the division of the apicoplast tubular network into individual small apicoplasts during sporozoite budding (Fig. 3A)^12^. These results clearly show that, in *P. berghei*, FabG is present in oocysts and oocyst sporozoites, which was unexpected in view of the reported absence of FabG expression in the same life cycle stages from the related parasite species *P. yoelii*, where FabG is first observed in the apicoplast of salivary gland sporozoites^3^. As FAS enzymes operate in a complex, we investigated if other components of this enzyme complex were expressed in *P. berghei* oocysts. To do so, we looked for the presence of mRNA specific for the genes encoding FabB/F, FabZ and FabI in unsporulated oocysts, using reverse transcription PCR^13^. Indeed, DNA fragments of the expected sizes were PCR-amplified for all four mRNA species investigated (including FabG-encoding mRNA as a positive control) (Fig. 3), confirming oocyst expression of all four FAS-specific genes. These results support the notion that *P. berghei* has an operational FAS apparatus throughout oocyst development, located in the apicoplast.

The reported redundant function of FabG—and hence of FAS—in oocysts^1^ was at first impression at odds with the oocyst-specific expression of FabG in *P. berghei* (Fig. 3). One possible explanation is that loss of FabG in these oocysts is compensated for by the other parasite-encoded ketoacyl reductase: KCR (PBANKA_0522400) that is normally part of FAE (Fig. 1). Notably, *P. berghei* KCR null mutant parasites are reported to produce oocysts that are incapable of forming sporozoites^1^, a phenotype that is very similar to that of FabI and FabB/F enzyme null mutants in *P. falciparum*^4^. To test this hypothesis, we generated a transgenic parasite line expressing KCR fused to GFP at its carboxy-terminus by allelic replacement of the wildtype *kcr* gene (Fig. 2C). Diagnostic PCR across the 3’-integration site amplified an approximately 2kb fragment of the expected size (Fig. 2D), confirming integration of the modified *kcr::gfp* allele into the target locus of the resulting KCR/GFP parasite line. Furthermore, the absence of the wildtype *kcr* allele in clonal populations of KCR/GFP was confirmed by diagnostic PCR (Fig. 2D).

**Figure 4:**
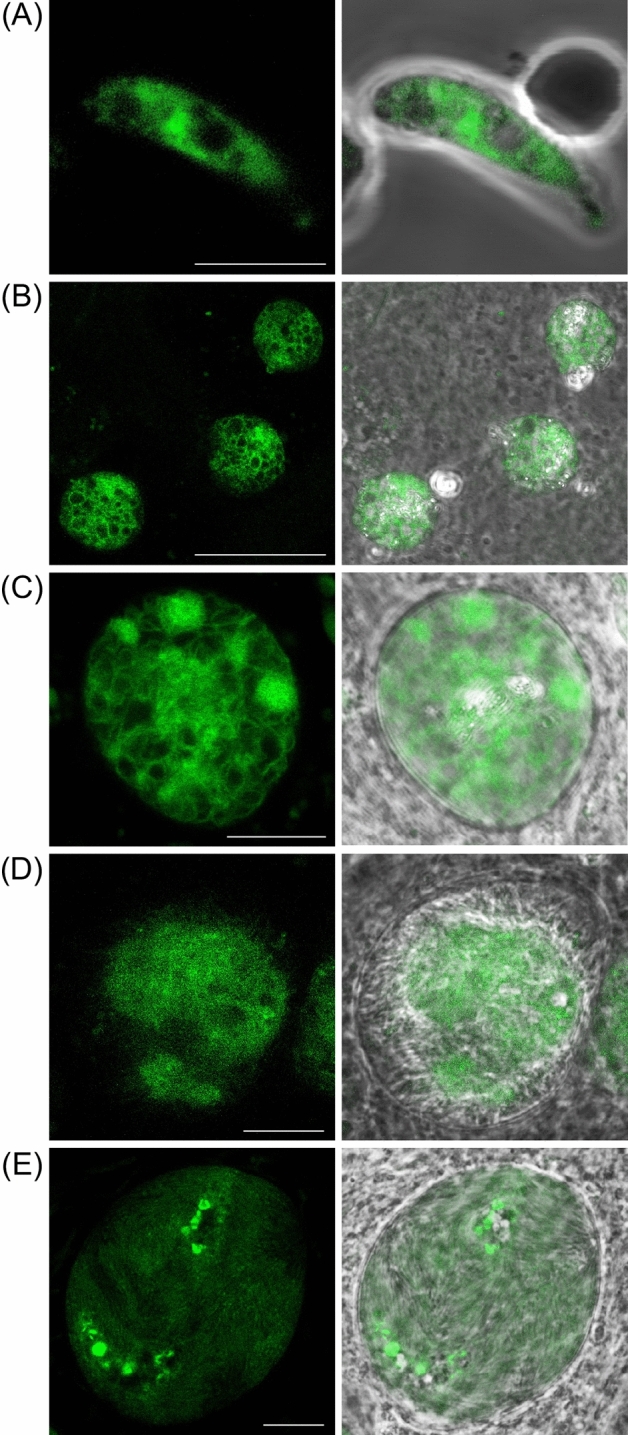
Expression of KCR in *P. berghei*. Confocal GFP fluorescence and brightfield images of parasite line KCR/GFP (clone 1), showing (**A**): a mature ookinete; (**B**): group of three young oocysts at 4 days post-infection; (**C**): an unsporulated oocyst at 8 days post-infection; (**D**) a sporulating oocyst at 14 days post-infection; (**E**) a fully sporulated oocyst at 14 days post-infection. Scale bar 10μm.

Reported transcript expression of the *kcr* gene in *P. berghei* blood stage parasites showed highest levels in female gametocytes^11,14^ and its mRNA is predicted to be subject to transcriptional repression^15^. These data were indicative of the KCR protein being expressed post-fertilisation. Consistent with this, live fluorescence microscopy of parasite line KCR/GFP revealed its expression in ookinetes (Fig. 4A). The KCR loss-of-function phenotype^1^ furthermore pointed to its expression in oocysts, and this was indeed confirmed by the presence of KCR::GFP-based fluorescence in these life stages (Fig. 4B,C). Parasite line KCR/GFP formed sporozoites and KCR::GFP-based fluorescence in budding sporozoites and in fully sporulated oocysts was notably weaker than in the oocyst sporoblast (Fig. 4D,E), indicating that KCR expression is downregulated, but not absent, in sporozoites. The normal sporozoite development in parasite line KCR/GFP indicates that the GFP fusion did not adversely affect KCR function, and hence the subcellular localisation of the protein. The localisation of KCR::GFP-based fluorescence was distinct from the putative apicoplast-specific localisation of FabG, and instead displayed a more widespread subcellular distribution consistent with a localisation in ER membranes (Fig. 4). This putative localisation is in agreement with the predicted structure of KCR, possessing an amino-terminal transmembrane helix and a carboxy-terminal di-lysine ER-retention signal in addition to its central enzymatic domain shared with FabG. The distinct subcellular localisation of KCR makes it unlikely that it can compensate for FabG loss in the reported FabG null mutant of *P. berghei*^1^, suggesting that there is no functional overlap between FAS and FAE in the oocyst. The *P. berghei* KCR null mutant phenotype reported^1^ therefore points to an essential role of FAE in sporozoite formation within the oocyst. A similar loss-of-function phenotype was reported for the FAE enzyme DEH (Fig. 1) in *P. berghei*^16^. Collectively, these observations indicate that *P. berghei* oocysts rely on FAE and are incapable of obtaining any or at least sufficient levels of long-chain FA from their environment.

The apparent difference in FabG expression between *P. berghei* (Fig. 3) and *P. yoelii*^3^ was surprising given the close evolutionary relationship between these two rodent malaria parasite species. It should be noted that different techniques were used to visualize FabG expression and localisation, namely GFP tagging combined with live fluorescence microscopy (*P. berghei*) and myc-tagging combined with immunofluorescence (*P. yoelii*), which may have contributed to these confounding results. Whereas the first approach leaves little room for false positive results, staining the internal oocyst by immunofluorescence is more challenging due to the oocyst capsule, which is poorly permeable to antibodies. Taken at face value, however, the results presented here reveal that *P. berghei* takes a position in between that of *P. yoelii* and *P. falciparum* with regards to FAS enzyme expression and reliance on FAS in the oocyst stages. Oocysts of *P. berghei* clearly can use FA scavenging from the mosquito haemolymph to support oocyst and sporozoite development in the absence of operational FAS^1^. Why then do *P. berghei* oocysts invest resources into the expression of an intact FAS system? One explanation is that FAS provides a selective advantage under conditions different from those used during analysis of the FabG null mutant phenotypes^1^. In this context it is important to note that, in experimental mosquito infections, the blood-fed insects are not allowed to lay eggs, resulting in recycling of nutrients through follicle resorption and higher nutrient/lipid availability for oocyst development than under natural conditions when eggs would normally be laid. Low haemolymph FA levels could also be reached in situations of high oocyst loads, when oocyst growth could potentially deplete FA resources before sporozoite budding commences. In such situations, FAS could kick in to promote/rescue sporozoite formation and ensure transmission. Indeed, *P. berghei* oocyst growth and sporogony are reduced by crowding, as well as by lowering lipid levels in the insect via lipophorin knockdown^5,6,17^. While the contribution of FAS to oocyst development and sporogony is clearly more important in *P. falciparum* than in *P. berghei*^1,4^, this may ultimately be a quantitative rather than a qualitative difference between these *Plasmodium* species that reflect biological differences for example in their efficacy of lipid uptake, or in their overall FA requirements.

The potential of *Plasmodium* oocysts to utilise FAS alongside FA scavenging—as revealed in this paper–is relevant to the non-competitive relationship of resource exploitation that has been proposed to exist between *Plasmodium* parasites and their mosquito vectors, and which shapes parasite virulence in both the insect and host^5,7^. Mosquito lipid trafficking by lipophorin is an important factor in this process^5,7^. This non-competitive strategy to optimise transmission while minimising the reproductive fitness cost to the insect can be achieved by the parasite using ‘excess’ nutritional resources not invested in oogenesis, or by using such resources only after completion of egg formation^5,7^. The ability of the parasite to switch to FAS could be an important additional contributor. Higher availability of lipids could stimulate FA scavenging, while lower lipid levels could stimulate FAS, in each case promoting sporogony without adversely affecting mosquito fitness, and allowing the parasite to fine-tune its relationship with the mosquito for exploiting FA-based nutrients to evolve optimal transmission. In any event, access to FA via both synthesis and scavenging should give the *Plasmodium* oocyst greater versatility to cope with environmental changes within the vector.

## Methods

### Ethics statement

As described previously^10^, all laboratory animal work was carried out in accordance with the United Kingdom Animals (Scientific Procedures) Act 1986 implementing European Directive 2010/63 for the protection of animals used for experimental purposes and was approved by the London School of Hygiene & Tropical Medicine Animal Welfare Ethical Review Body and United Kingdom Home Office. Experiments were typically conducted in 6–8 weeks old CD1 mice, specific pathogen free and maintained in filter cages, following ARRIVE guidelines. Animal welfare was assessed daily and upon reaching experimental or clinical endpoints animals were humanely euthanized by exposure to carbon dioxide gas in a rising concentration. Mice were infected with parasites suspended in phosphate buffered saline (PBS) by intraperitoneal injection, or by infected mosquito bite on anaesthetized animals. Intra-erythrocytic parasitemia was monitored regularly by microsampling blood from a superficial tail vein. Drugs were administered by intraperitoneal injection or where possible were supplied in drinking water. Parasitized blood was harvested by cardiac bleed under general anaesthesia without recovery.

### Parasite maintenance, culture and transmission

*P. berghei* ANKA clone 2.34 parasites were maintained as cryopreserved stabilates or by mechanical blood passage and regular mosquito transmission, as previously described^10^. Mosquito infection and transmission assays were as previously described using *Anopheles stephensi*^18,19^ and infected insects were maintained at 20°C at approximately 70% relative humidity under a 12h/12h light/dark cycle. Ookinete cultures were set up overnight from gametocytemic blood as described^20^.

### Generation of transgenic parasite lines

To generate a parasite line expressing FabG fused to GFP (FabG/GFP), an approximately 2.2kb fragment corresponding to the coding sequence (plus introns) and 5’UTR of PBANKA_0823800 was PCR-amplified with primers FabG-F (TTGGGCTGCAGTCGAGGAATTTCCATAGCATCCATATATAC) and FabG-R (AATGAGGGCCCCTAAGCTGGTACCACTTGATAATCCACCATCAATTATAAATAC) and cloned into *Sal*I/*Hin*dIII digested pBS-EGFP-hDHFR^10^ by In-Fusion to give plasmid pBS-FabG/GFP. This plasmid was linearised with *Pac*I and transfected into purified schizonts for integration into the *fabg* locus by single crossover homologous recombination (Fig. 2A).

To generate a parasite line expressing KCR fused to GFP (KCR/GFP), the coding sequence of PBANKA_005524 plus approximately 0.45 kb of the 5′UTR was PCR amplified with primers pDNR-05524-F (ACGAAGTTATCAGTCGA*GGTACC*TGATTCAACTATATTACCGCAGATAC) and pDNR-05524-R (ATGAGGGCCCCTAAGCTTTCTTCTTTCTTCATTTTTTTCAAC) and introduced into *Sal*I/*Hin*dIII-digested pDNR-EGFP via In-Fusion cloning to give plasmid pDNR-KCR/EGFP. An approximately 0.77 kb sequence corresponding to the 3′UTR of PBANKA_005524 was PCR amplified with primers pLP-05224-F (ATATGCTAGAGCGGCCAGTGTGCTCACTTTTTGTTATTTTG) and pLP-05224-R (CACCGCGGTGGCGGCCTTTGGAAAATATGCAAAGC) and introduced into *Not*I-digested pLP-hDHFR via In-Fusion cloning to give plasmid pLP-hDHFR/KCR. The *kcr*-specific sequence from pDNR-KCR/EGFP was introduced into pLP-hDHFR/KCR via Cre-*loxP* site-specific recombination to give the final construct pLP-KCR/EGFP. This plasmid was linearised with *KpnI* and *Sac*II and transfected into purified schizonts for integration into the *kcr* locus by double crossover homologous recombination (Fig. 2C).

Parasite transfection, pyrimethamine selection and limiting dilution cloning were performed as described^21,22^.

### Diagnostic PCR

Genomic DNA extraction for diagnostic PCR was performed as described^19^. To confirm integration of the modified GFP-tagged *fabg* allele into the target locus, genomic DNA of the FabG/GFP parasite line was subjected to diagnostic PCR with primers P1 (ATGTGCAGTGATTTTGCATATCT) and P2 (GTGCCCATTAACATCACC) to amplify an approximately 2.4kb fragment across the 5’-integration site (Fig. 2A,B). The absence of the parental unmodified *fabg* allele (2.9kb) was assessed by PCR with primers P1 and P3 (CACCGCGGTGGCGGCCCTTATAAGTATTCTTCTCATATTTCCCGT) (Fig. 2A,B).

To confirm integration of the GFP-tagged *kcr* allele into the target locus, genomic DNA of the KCR/GFP parasite line was subjected to diagnostic PCR with primers P4 (ACAAAGAATTCATGGTTGGTTCGCTAAACT) and P5 (CATTAGCATTAGGTTTCATTTCATTAC) to amplify an approximately 2.0kb fragment across the 3’-integration site (Fig. 2C,D). The absence of the parental *kcr* allele (3.0kb) was assessed by PCR with primers P6 (ACGAAGTTATCAGTCGA*GGTACC*TGATTCAACTATATTACCGCAGATAC) and P5 (Fig. 2C,D).

Images of full-size DNA agarose gels with complete DNA ladders are included in the Supplementary Information file, with regions used in Fig. 2 denoted with red boxes.

### Reverse transcription PCR

As described previously^13^, approximately 30 *An. stephensi* oocyst-containing midguts taken seven days after a wildtype *P. berghei*-infected blood meal were dissected and pooled, and total RNA was extracted using the RNeasy mini kit (Qiagen). To remove residual genomic DNA contamination, RNA samples were subjected to DNase I treatment (amplification grade, Sigma) for 15min at room temperature followed by heat inactivation of the enzyme. After a second RNeasy clean-up step to remove the DNase I, RNA samples were reverse transcribed with Moloney murine leukemia virus reverse transcriptase (RNaseH minus, point mutation, Promega) in the presence of oligo(dT)_25_ at 50 °C for 1h, followed by heat inactivation. The resultant cDNA samples were column purified using the QIAquick gel extraction kit (Qiagen) and eluted in water. Fab enzyme-specific cDNA was PCR amplified (25 cycles) with primer pairs CATAATCTGAATAAGGTTAATCCAAATAAG and TACCATATTTGCAATCTCTTCTGG (FabG, fragment size 769bp); GAAGATAACATGTTTCACTAACAATGG and ATACCACCACCATATCCAGGTAC (FabI, fragment size 750bp); TTTTTGGTTCACCAAGTGTATAGC and GCGCATGCACTTAACATACC (FabB/F, fragment size 639bp); GAAACTTTTTATAATTTTTGTTTACGTCC and CACTAATGTATCGCCTGGTAAAAC (FabZ, fragment size 562bp). These primers were designed to encompass an intron splice site, where possible, to ensure mRNA specific amplification. An image of the full-size DNA agarose gels with complete DNA ladder is included in the Supplementary Information file.

### Microscopy

Live parasite samples were assessed, and images captured, on a Zeiss LSM880 laser scanning confocal microscope using 100 × oil objective and ZEN Blue version 3.0 software, as described^10^. Protein expression and subcellular localisation were assessed and compared in oocysts from mosquito batches infected with uncloned and cloned populations of transgenic parasites to ensure results were consistent and representative.

### Supplementary Information

Supplementary Information.

## Data Availability

All data generated or analysed during this study are included in this published article and its Supplementary Information files.
