# Supplementary material for: Plasmodium berghei oocysts possess fatty acid synthesis and scavenging routes
Source: Sci Rep. 2023 Aug 5;13:12700. doi: 10.1038/s41598-023-39708-z (PMC10404217; doi:10.1038/s41598-023-39708-z)

Full-length DNA agarose gel corresponding to Fig. 2B

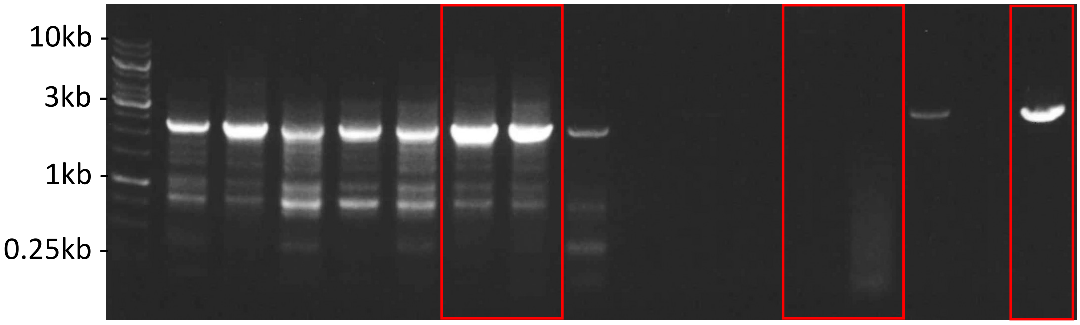

Full-length DNA agarose gel corresponding to Fig. 2D

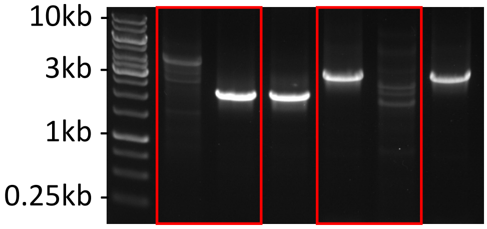

Full-length DNA agarose gel corresponding to Fig. 3B

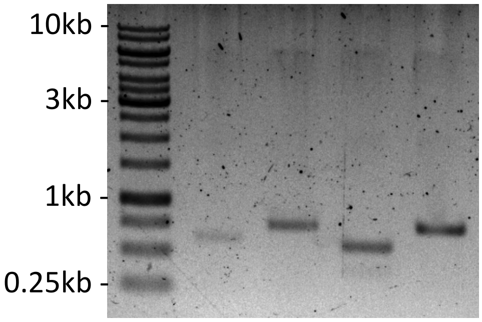

Supplement: Supplementary file 1 — Supplementary Information. [file 41598_2023_39708_MOESM1_ESM.pdf]
